# Downregulation of microRNA-21 inhibited radiation-resistance of esophageal squamous cell carcinoma

**DOI:** 10.1186/s12935-018-0502-6

**Published:** 2018-03-20

**Authors:** Fang Li, Jia-Hua Lv, Long Liang, Jun-chao Wang, Chu-Rong Li, Lei Sun, Tao Li

**Affiliations:** Department of Radiation Oncology, School of Medicine, Sichuan Cancer Hospital & Institute, and Sichuan Cancer Center, University of Electronic Science and Technology of China, No. 55, 4th Section of Renmin South Road, Chengdu, 610041 Sichuan China

**Keywords:** miR-21, ESCC, Radiation-resistance, Tumor stage

## Abstract

**Background:**

MicroRNA-21 (miR-21) was previously reported being dysregulated in many kinds of cancer including esophageal squamous cell carcinoma (ESCC). In the present study, we aimed to investigate the role of miR-21 in ESCC, especially in its effects on radiation-sensitivity of ESCC.

**Methods:**

Expression of miR-21 was detected in 63 pairs ESCC tumor and adjacent non-tumoral tissues using qRT-PCR, correlation between miR-21 and clinicopathological feature of ESCC was analyzed. The role of miR-21 in the proliferation, cell cycle and apoptosis of ESCC cells during irradiation were studied.

**Results:**

MicroRNA-21 expression was significantly increased in ESCC tumor tissues. Expression of miR-21 was positively associated with advanced clinical stage. Under irradiation, overexpression of miR-21 increased cell proliferation and cells in S phase, and inhibited cell apoptosis of ESCC cells. In contrast, knockdown of miR-21 had an opposite effect.

**Conclusions:**

Downregulation of miR-21 inhibited the radiation-resistance of ESCC, whereas overexpression of miR-21 increased the radiation-resistance. MiR-21 is a potential novel target for developing specific treatment interventions in ESCC in future.

## Background

Esophageal squamous cell carcinoma (ESCC) is the most common esophageal cancer and also the most leading cause of cancer-related death worldwide [1, 2]. Nowadays, the incidence and mortality rate of ESCC were increased rapidly in China, with three times in men than women [3, 4]. Although the treatment and therapy of ESCC have been improved by surgical resection and radiochemotherapy, its prognosis remains not satisfying [5–8]. Therefore, fully understanding the exact molecular mechanism in ESCC progression is urgent for researchers to develop diagnostic and therapy strategies for ESCC.

MicroRNA (miRNA) is a class of small, evolutionary conserved RNA molecules that suppresses gene expression at the post-transcriptional level [9, 10]. Our understanding on the mechanism that negatively regulated gene expression was broadened by the discovery of these small non-coding transcripts, which added an entirely novel field of regulatory mechanism of cellular function. MiRNAs act by base-pairing with their target mRNAs through complementarity at the 3′ untranslated regions (UTRs) of the target mRNAs [9, 10]. This results in repression or direct cleavage of their translations [9, 10]. However, in some cases, mRNA translation was enhanced by miRNAs. For example, miR-10a could bind the 5′-UTR of mRNAs of ribosomal protein enhancing their translation [11, 12]. Moreover, some miRNAs could switch their roles from translation repression to promotion in a cell cycle-dependent manner. It was reported a number of miRNAs play important roles in cell proliferation, apoptosis, and differentiation, so as to involve in normal physiological and pathological conditions [9, 13]. miRNAs also contributed to oncogenesis by suppressing tumor suppressor genes or by promoting the expression of oncogenes. Thus, some miRNAs were identified as cancer diagnostic and prognosic makers [9].

MiRNA-21 is one of the miRNAs to be identified as transcribed by RNA polymerase II. MiR-21 has been identified as a major driver of miRNA transcription [14, 15]. The gene coding for pri-miR-21 is located within the intronic region of the TMEM49 gene. Despite pri-miR-21 and TMEM49 are overlapping genes in the same direction of transcription, pri-miR-21 is independently transcribed by its own promoter regions and terminated with its own poly (A) tail. After transcription, pri-miR-21 is finally processed into mature miR-21 [16]. It was demonstrated that miR-21 overexpressed in multiple malignancies, including renal carcinoma, breast cancer, ovarian cancer, non-small cell lung cancer, gastric cancer, colon cancer, and esophageal cancer [17–23]. MiR-21 functioned as an oncogene and associated with tumor aggression and carcinogenesis, which in partly mediated by preventing apoptosis [24, 25]. In the present study, we examined the expression of miR-21 in ESCC and investigated its effects on radiation-sensitivity of ESCC. The results indicated that miR-21 was overexpressed in ESCC, and the downregulation of miR-21 could suppress the radiation-resistance of ESCC.

## Materials and methods

### Patients and specimens

Sixty-three paired ESCC tissues and adjacent non-tumoral tissues were collected from patients who were diagnosed as ESCC at Sichuan Cancer Hospital & Institute, and Sichuan Cancer Center, School of Medicine, University of Electronic Science and Technology of China. Primary tumor regions and corresponding histologically non-tumoral tissues (within 2 cm far from the tumor) from the same patients were separated by experienced pathologists, and immediately stored in liquid nitrogen (− 193 °C) until use. Before surgical resection preoperative, none subjects have received treatments of ESCC such as chemotherapy or radiotherapy. All patients were signed informed consent forms before sample collection. A comprehensive set of clinicopathological data including age, gender, size of primary tumor, tumor differentiation and TNM stage were recorded, which are summarized in Table 1. The stage of disease was determined according to the clinical TNM classification system. Use of patient samples was approved by the ethics committee of our Institute.

**Table 1:** Relationship between miR-21 level and clinicopathologic characteristics in ESCC patients

Clinicopathologic characteristics	Number of cases	MiR-21 expression		*P*
		Low-miR-21 (n = 20)	High-miR-21 (n = 43)	
Age				
≥ 60	45	11	28	0.213
< 60	18	9	15	
Gender				
Male	34	12	22	0.303
Female	29	8	21	
Size of primary tumor				
< 5 cm	29	9	20	0.538
≥ 5 cm	34	11	23	
Tumor differentiation				
Highly differentiated	26	7	19	0.472
Moderately differentiated	26	7	19	
Low differentiated	11	6	5	
TNM stages				
I + II	29	16	13	0.004
III + IV	34	4	30	

*TNM* tumour, node and metastasis

### Cell culture and ionizing radiation

The human ESCC cell lines TE-1, EC1 and KYSE140 were purchased from the Chinese Academy of Sciences, Shanghai, China. Cells were cultured in RPMI 1640 media supplemented with 10% fetal bovine serum (HyClone, USA), 100 U/ml penicillin and 100 µg/ml streptomycin, and maintained at 37 °C in 5% CO_2_. All experimental cells were in the logarithmic growth phase. Cells were exposed to the indicated dose of irradiation in a JL Shepherd Model 143 ^137^Cesium γ-irradiated at a rate of 2.4 Gy/min.

### RNA isolation and quantitative real-time PCR (qRT-PCR)

Total RNAs were isolated from 63 pairs ESCC tumor and adjacent non-tumoral tissues, and ESCC cells with or without irradiation using the Absolutely RNA™ RT-PCR Miniprep Kit (Stratagene, China). For reverse transcription (RT) reactions, 10 ng total RNA was used in each reaction (5 μl) and mixed with RT primer (3 μl). For qRT-PCR, expression of mature miR-21 was detected using TaqMan miRNA assays (ABI PRISM) with the stem-loop method. PCR was conducted by ABI 7500 Real-time PCR system (ABI, USA). The primers used in this study were listed: miR-21-5p: UAGCUUAUCAGACUGAUGUUGA, miR-21-5p RT: CTCAACTGGTGTCGTGGAGTC GGCAATTCAGTTGAGTCAACATC, miR-21-5p F: ACACTCCAGCTGGGTAGCTTATCAG ACTGATG, All R: CTCAACTGGTGTCGTGGA, U6 F: CTCGCTTCGGCAGCACA, U6 R: AACGCTTCACGAATTTGCGT. U6 was used for normalization. Relative expression levels were calculated using the 2^−ΔΔCt^ method.

### MiRNA mimics or inhibitors transfection

MiR-21 inhibitors or mimics vector was constructed. A scrambled sequence without significant homology to any rat, mouse or human gene was used as a negative control (NC group). Transfection was performed using Lipofectamine™ 2000 (Invitrogen, USA) according to the manufacturer’s manual. After transfection 48 h, miR-21 expression was assessed by qRT-PCR.

### Cell proliferation assay

After transfection 48 h, the effects of miR-21 expression on TE-1 cell proliferation were assessed using the Cell Counting Kit-8 (CCK-8, Beyotime, China). The cells were plated in 96-well plates and irradiated with 6 Gy γ-ray. After 1, 2, 3, and 4 days, CCK-8 (10 μl) was added and incubated at 37 °C for 1.5 h, respectively. Finally, cell viability were measured using a microplate reader at 450 nm.

### Cell apoptosis and cell cycle

Cell cycle and apoptosis were detected using flow cytometry. Cells were harvested after irradiation of 6 Gy for 8 h. Briefly, cell monolayers were washed with PBS, trypsinzed and resuspended in ice-cold PBS. Cells were fixed in 70% ethanol and incubated with propidium iodide (PI) staining solution for 30 min in the dark. The fluorescence was measured using a Flow Cytometer (BD, USA) and cell cycle distribution was analyzed. For apoptosis detection, the annexin V-FITC apoptosis detection kit (BD, USA) was used according to the manufacturer’s instructions.

### Western blot

The western blot was performed for protein detection. Total proteins were extracted, separated by 10% SDS-polyacrylamide gel electrophoresis (SDS-PAGE), and transferred to the PVDF. The blot was incubated with primary antibody against cleaved-PARP and cleaved-caspase 3 (1:1000, Cell Signaling Technology, USA) overnight at 4 °C, and incubated with HRP-conjugated secondary antibody for 1 h. GAPDH antibody was used as control. Finally, ECL was used to visual the blots.

### Statistical analyses

The data was presented as mean ± standard deviation (SD). All statistical analyses were performed using SPSS software 15.0 (SPSS, USA) in at least three independent experiments. Two-tailed Student’s t test or One-way ANOVA was used to analysis the differences among the groups. *P* < 0.05 was considered as statistical significance.

## Results

### MiR-21 was upregulated in ESCC tissue compared with the non-tumoral tissue

**Fig. 1:**
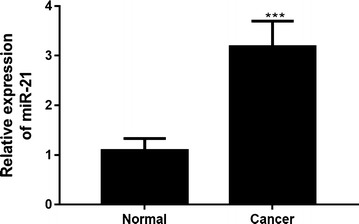
Expression of miR-21 in esophageal squamous cell carcinoma. 63 pairs of esophageal squamous cell carcinoma and adjacent non-tumoral tissues were collected, and the expression of miR-21 was detected by qRT-PCR. ***P < 0.001

Expression of miR-21 was detected in 63 pairs ESCC and adjacent non-tumoral tissues using qRT-PCR. Results showed miR-21 levels were significantly higher in ESCC tissues than that in corresponding non-cancerous tissues (68.3%, 43/63) (Fig. 1). Next, we evaluated the correlation between levels of miR-21 and clinicopathological features of ESCC patients. Remarkably, as showed in Table 1, a significant association existed between miR-21 and TNM stage, indicating patients with high miR-21 level was significantly correlated with advanced TNM stage in ESCC.

### MiR-21 was radiation-sensitivity in ESCC cells

**Fig. 2:**
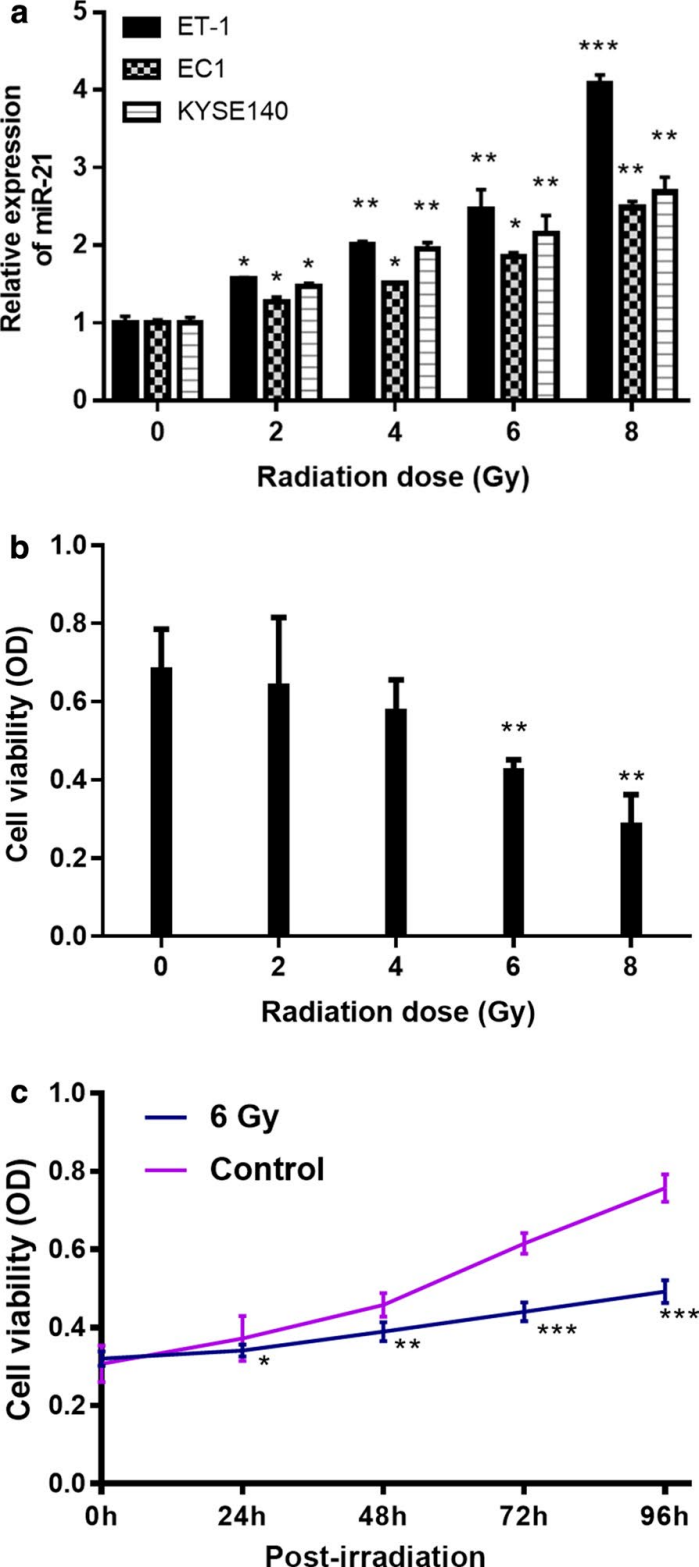
Effect of different radiation dose on level of miR-21 and cell viability in TE-1 cells. Cells were exposed to different dose (0, 2, 4, 6, 8 Gy) of irradiation at a rate of 2.4 Gy/min and subsequently cultured for 48 h. Then, cells were collected. **a** miR-21 level and **b** cell viability were detected by qRT-PCR and MTT assay, respectively. **c** After irradiation at 6 Gy, cell viability in TE-1 cells was detected. *P < 0.05, **P < 0.01, ***P < 0.001 vs. 0 Gy/min

MiR-21 expression was examined in TE-1, EC1 and KYSE140 cells after radiation with a dose of 0, 2, 4, 6 and 8 Gy. MiR-21 expression was increased with radiation dose in all these cells (Fig. 2a), indicating miR-21 was radiation-sensitivity in ESCC cells. Among them, the miR-21 in TE-1 cells were most increased (Fig. 2a), indicating that TE-1 was most sensitivity to irradiation among them. Thus, subsequent experiments were studied using TE-1 cells.

Cell viability was performed (Fig. 2b and c). Results showed that radiation at 6 and 8 Gy significantly inhibited the cell viability in TE-1 cells (Fig. 2b). After 6 Gy irradiation, cell viability was significantly inhibited at 24, 48, 72, and 96 h (Fig. 2c). These findings suggest 6 Gy at 24 h inhibited cell viability of TE-1 cells.

### Knockdown or overexpression of miR-21 in ESCC cells

**Fig. 3:**
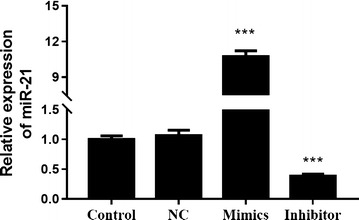
Expression of miR-21 after transfection of miR-21 mimics and inhibitor in TE-1 cells. After transfection 48 h, expression of miR-21 was detected by qRT-PCR. ***P < 0.001

To determine the role of miR-21 in ESCC under irradiation, TE-1 cells were transfected with miR-21 mimics, inhibitor or NC vector. After transfection 48 h, the downregulation or overexpression of miR-21 in cells were confirmed (Fig. 3). Then, these cells were used for the subsequent experiments.

### Knockdown of miR-21 suppressed cell proliferation in TE-1 cells under irradiation, while overexpression increased cell proliferation

**Fig. 4:**
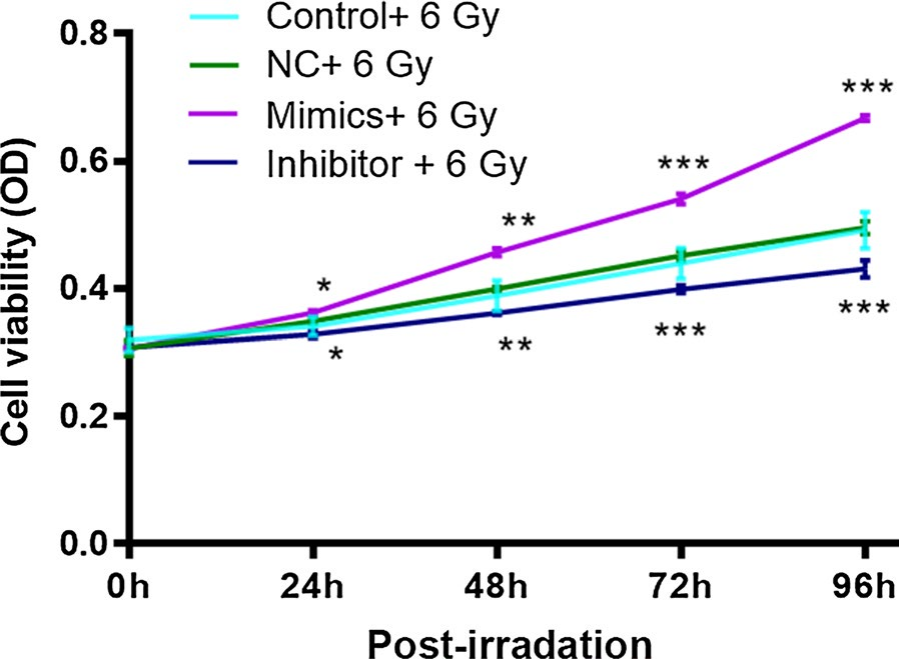
Cell viability of miR-21 mimics and inhibitor transfected-TE-1 cells after exposure of 6 Gy for 48 h. After transfection with miR-21 mimics and inhibitor, cells were irradiated with 6 Gy for 48 h. And then, the cell viability was detected by CCK8 assay

After transfection 48 h, cells were exposed to irradiation of 6 Gy at a rate of 2.4 Gy/min for 48 h. Then, cell proliferation assay was performed (Fig. 4). Results showed that knockdown of miR-21 significantly inhibited cell viability compared with NC and control under irradiation. In contrast, overexpression of miR-21 significantly increased cell viability compared with NC and control under irradiation. Thus, although radiation inhibited cell viability (Fig. 2c), the miR-21 levels influenced the effect of radiation on cell proliferation in TE-1 cells.

### Knockdown and overexpression of miR-21 affected the cell apoptosis and cell cycle distribution of TE-1 cells under gamma radiation

**Fig. 5:**
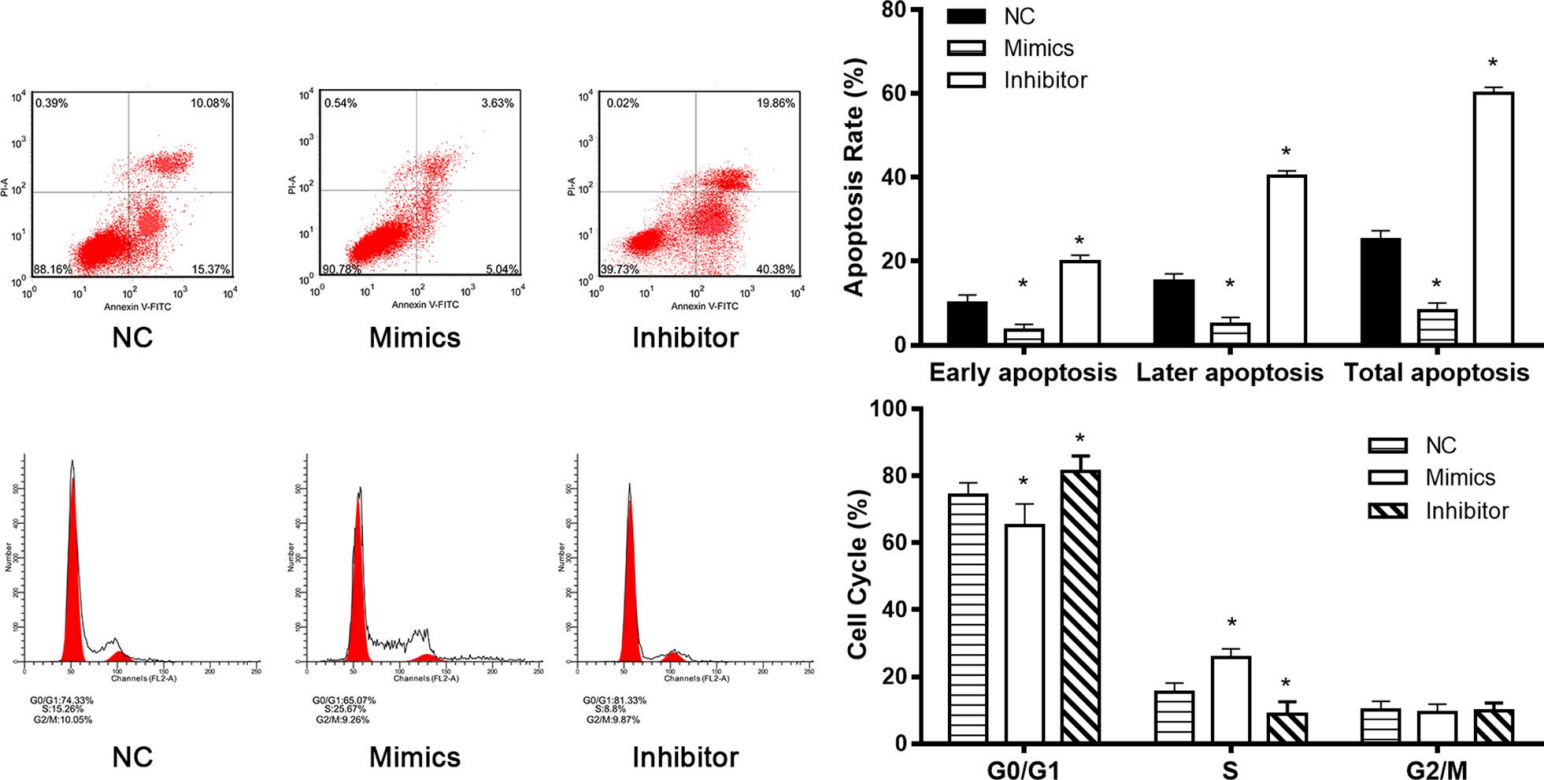
Cell apoptosis and cell cycle of miR-21 mimics and inhibitor transfected-TE-1 cells after exposure of 6 Gy for 48 h. After transfection with miR-21 mimics and inhibitor, cells were irradiated with 6 Gy for 48 h. And then, the cell apoptosis and cell cycle were detected by flow cytometry

Cell apoptosis analysis was carried out to determine whether the effect of miR-21 on cell proliferation was due to cell apoptosis alterations. After transfection 48 h, cells were exposed to 6 Gy γ-ray for 48 h. Then, these cells were harvested and cell apoptosis was examined by flow cytometry (Fig. 5). Under irradiation, the percentage of apoptosis cells in miR-21 knockdown group was significantly higher than NC, while that in miR-21 overexpression group was significantly lower than NC.

We next explored role of miR-21 in cell cycle distribution of ESCC cells under irradiation. After transfection 48 h, cells were exposed to 6 Gy γ-ray for 48 h. Then, cell cycle was examined by flow cytometry (Fig. 5). The results showed that the percentage of cells in G1/G0 phase in cells transfected with miR-21 inhibitor was significantly increased compared with NC, with significantly decrease in the population of cells in S phase. In contrast, the percentage of cells in G1/G0 phase was significantly decreased in miR-21 overexpression group compared with NC, with significantly increased in the population of cells in S phase (Fig. 5). These results suggested that downregulation of miR-21 induced cell arrest in G1/GO in TE-1 cells, but the overexpression of miR-21 increased cells in S phase.

**Fig. 6:**
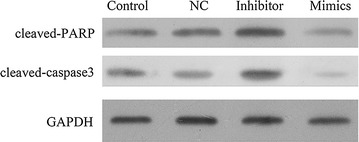
Expression of cleaved-caspase-3 and cleaved-PARP in miR-21 mimics and inhibitor transfected-TE-1 cells after exposure of 6 Gy for 48 h. After transfection with miR-21 mimics and inhibitor, cells were irradiated with 6 Gy for 48 h. And then, the cells were collected and the levels of cleaved-caspase-3 and cleaved-PARP were detected by western blot

We then examined the effects of miR-21 knockdown or expression on caspase 3 and PARP activities (apoptosis marker) after 6 Gy irradiation for 48 h. MiR-21 knockdown markedly increased the PARP activity and caspase 3 activity, compared with control or NC, while overexpression of miR-21 significantly decreased the PARP activity and caspase 3 activity (Fig. 6).

## Discussion

Studies have demonstrated that miRNAs levels are related to clinical and biological characteristics of tumors [14, 16]. The miRNAs have potential diagnostic value. MiRNAs play important roles in cancer progression [9]. For example, miR-21 is an oncogenic miRNA. Overexpression of miRNA-21 promoted the development and progression of various cancers. MiR-21 level was significantly elevated in both tissue and serum samples of esophageal cancer patients [27]. It was demonstrated that overexpression of miR-21 was an independent negative prognostic factor for overall survival in ESCC patients [26, 27]. MiR-21 had higher expression in esophageal cancer tissues with lymph node metastasis and poor differentiation [26]. Metastasis is the most deadly and least understood aspect of cancer [28]. In the present study, although we did not detect metastasis, we found that the miR-21 levels were significantly higher in ESCC tumor tissues and existed a significant association with TNM stage, indicating patients with high miR-21 expression level was significantly correlated with advanced TNM stage in ESCC patients. It was suggested that miR-21 might be a useful diagnostic and prognostic marker in ESCC patients.

Although many studies have explored the role of miR-21 in cancer, only few reports studied the relationship between miR-21 expression and radiation-sensitivity of cancer. Yu et al. [29] detected the plasma miRNAs that associated with carcinogenesis or radiobiology of ESCC. They found miR-21 expression was significantly inhibited in radiotherapy sensitive patients. It was reported that miR-21 was the only one that increased sixfolds in high-low linear energy transfer radiation promoted mouse liver tumors, compared with that in the nonirradiated liver tissues [30]. MiR-21 was overexpression in human or mouse hepatocytes after exposure to radiation, as well as in liver tissues derived from whole body irradiated mice [30]. Thus, miR-21 may play a role in the radiation-sensitivity of tumors. In the present study, considering the radiation therapy is one of the most important treatment for ESCC, we examined the effects of miR-21 expression on radiation-sensitivity of ESCC cells. Results showed that miR-21 was significantly increased in ESCC cell lines after irradiation, suggesting miR-21 might contribute in evading cancer therapy. The results showed that under irradiation, downregulation of miR-21 inhibited cell proliferation, promoted cell apoptosis and cell arrest in GO/G1 phase, suggesting downregulation of miR-21 promoted the radiation-sensitivity of TE-1 cells. In contrast, the overexpression of miR-21 had the opposite effects. We did not further explore the potential mechanism. But it had shown miR-21 had the ability to suppress the expression of the tumor suppressor PTEN and PDCD4 [26, 31]. These mechanisms may also be function in the condition of irradiation. This should be investigated in future. At the molecular level, cleaved-caspase-3 and cleaved-PARP were increased in miR-21 knockdown cells under irradiation. In cultured glioblastoma cells, downregulation of miR-21 triggered activation of caspases leading to increased cell apoptosis [25]. The findings suggested that dysregulation of miR-21 might associate with development and radiation-resistance of cancer via regulating the cell apoptosis.

## Conclusion

In conclusion, our results demonstrated an overexpression of miR-21 in ESCC tumors. Overexpression of miR-21 was strongly associated with advanced TNM stage. In addition, Downregulation of miR-21 inhibited the radiation-resistance. In contrast, overexpression of miR-21 had an opposite effect. Our findings provide a novel role of miR-21 in ESCC, which might suggest a promising novel target for developing the therapy strategies in ESCC.

## Abbreviations

miR-21: MicroRNA-21
ESCC: esophageal squamous cell carcinoma
UTRs: untranslated regions
CCK8: Cell Counting Kit-8
SDS-PAGE: SDS-polyacrylamide gel electrophoresis
RNU6B: 6B small nuclear RNA gene
